# A phenotypic high-content, high-throughput screen identifies inhibitors of NLRP3 inflammasome activation

**DOI:** 10.1038/s41598-021-94850-w

**Published:** 2021-07-28

**Authors:** Sohaib Nizami, Val Millar, Kanisa Arunasalam, Tryfon Zarganes-Tzitzikas, David Brough, Gary Tresadern, Paul E. Brennan, John B. Davis, Daniel Ebner, Elena Di Daniel

**Affiliations:** 1 Alzheimer’s Research UK Oxford Drug Discovery Institute, NDM Research Building, University of Oxford, Old Road Campus, Roosevelt Drive, Oxford, OX3 7FZ UK; 2 Target Discovery Institute, NDM Research Building, University of Oxford, Old Road Campus, Roosevelt Drive, Oxford, OX3 7FZ UK; 3 Division of Neuroscience and Experimental Psychology, School of Biological Sciences, Faculty of Biology, Medicine and Health, Manchester Academic Health Science Centre, University of Manchester, AV Hill Building, Oxford Road, Manchester, M13 9PT UK; 4 Lydia Becker Institute of Immunology and Inflammation, University of Manchester, Manchester, M13 9PT UK; 5 National Phenotypic Screening Centre, Target Discovery Institute, NDM Research Building, University of Oxford, Old Road Campus, Roosevelt Drive, Oxford, OX3 7FZ UK; 6 Janssen Research and Development, Turnhoutseweg 30, 2340 Beerse, Belgium

**Keywords:** Diseases of the nervous system, Cellular neuroscience, Neurological disorders, Drug discovery, Business strategy in drug development, Drug screening, Pharmacology, Target validation

## Abstract

Inhibition of the NACHT, LRR and PYD domains-containing protein 3 (NLRP3) inflammasome has recently emerged as a promising therapeutic target for several inflammatory diseases. After priming and activation by inflammation triggers, NLRP3 forms a complex with apoptosis-associated speck-like protein containing a CARD domain (ASC) followed by formation of the active inflammasome. Identification of inhibitors of NLRP3 activation requires a well-validated primary high-throughput assay followed by the deployment of a screening cascade of assays enabling studies of structure–activity relationship, compound selectivity and efficacy in disease models. We optimized a NLRP3-dependent fluorescent tagged ASC speck formation assay in murine immortalized bone marrow-derived macrophages and utilized it to screen a compound library of 81,000 small molecules. Our high-content screening assay yielded robust assay metrics and identified a number of inhibitors of NLRP3-dependent ASC speck formation, including compounds targeting HSP90, JAK and IKK-β. Additional assays to investigate inflammasome priming or activation, NLRP3 downstream effectors such as caspase-1, IL-1β and pyroptosis form the basis of a screening cascade to identify NLRP3 inflammasome inhibitors in drug discovery programs.

## Introduction

Inflammation is an important protective mechanism in response to harmful stimuli, however it can be detrimental if uncontrolled. The over-production and release of proinflammatory cytokines, such as interleukin-1β (IL-1β), can initiate or propagate pathologies, such as immunological diseases and neurodegeneration^1^. The inflammasome is a major regulator of IL-1β production^1^. There are numerous types of inflammasomes with variable expression levels across tissues, with a primary example being NLRP3. NLRP3 is abundant in microglial cells^2^, which mediate the immune responses in the brain by helping to clear cell debris and dead neurons but have been implicated in the pathophysiology of neurodegeneration^3^. Priming of the canonical NLRP3 inflammasome response is initiated by pathogen-associated molecular patterns (PAMPs), molecular motifs found in bacteria, such as lipopolysaccharide (LPS), or damage-associated molecular patterns (DAMPs), which activate Toll-like receptors (TLRs)^4^ leading to the translocation of NF-кB from the cytosol to the nucleus increasing expression of NLRP3 and pro-IL-1β. NLRP3 priming can also be mediated by cytokine signaling, such as IL-1β or TNF-α, it is not limited to TLR-mediated priming. NLRP3 activation can be triggered by a plethora of diverse stimuli, including potassium ionophores, which mediate potassium efflux from the cells potentially via the involvement of NEK7^5–7^. NLRP3 oligomerizes and binds to ASC, and ASC binds to pro-caspase-1^2,3^, forming a multi-protein complex. Active caspase-1 cleaves pro-IL1β and pro-IL-18 to mature IL-1β and IL-18, which are released into the extracellular space, and can also induce a form of cell death called pyroptosis, via cleavage of gasdermin D^8–10^.

Small molecule inhibitors of the NLRP3 inflammasome have the potential to decrease inflammation and inflammasome-mediated cell death. MCC950 (also known as CRID3, and CP-456773) is a potent (nM) direct inhibitor of NLRP3, which closes the active NLRP3 conformation into an inactive state^11,12^. MCC950 inhibits IL-1β production in in vitro and in vivo models where it attenuates the severity of autoimmune encephalomyelitis^13–17^ and protects from dopaminergic neuron loss and motor dysfunction in toxin and genetic models of Parkinson’s disease^18^. Furthermore, NLRP3 inflammasome inhibition by MCC950 has shown therapeutic benefits in an age-related metabolic syndrome model^19^. MCC950 has not progressed through clinical trials due to reported liver toxicity^20^. There is therefore a great interest in developing small molecule NLRP3 inhibitors that are potent and safe in the clinic.

The aim of our study was to identify novel small molecule NLRP3 inflammasome inhibitors utilizing a robust phenotypic assay fit for drug screening. In the primary screen we used an immortalized cell line of immortalized bone marrow-derived macrophages (iBMDMs) with a fluorescent mCherry tag attached to the ASC protein allowing speck visualization after NLRP3 activation^21^. Here we describe a screening campaign carried out in collaboration with the National Phenotypic Screening Centre/Phenomics Discovery initiative (NPSC/PDi) whereby a diverse library and chemogenomic set of 81,000 small molecule compounds were screened in the speck assay resulting in the identification of several positive hits that decreased speck formation, including small molecule inhibitors of heat shock protein 90 (HSP90), Janus kinase (JAK) and IkB kinase (IKK-β). Selected hit molecules were also tested in other NLRP3 downstream cellular assays, such as IL-1β release, caspase-1 activation, and pyroptotic cell death.

## Results

### High-content imaging assay development

ASC speck assay development involved the optimization of several parameters including cell density, DMSO compatibility (as this was the vehicle used to solubilize the compounds), LPS and nigericin concentrations and incubation time (Figure S1). We identified the following optimal conditions: 10,000 cells per well to provide an optimal monolayer and enough number of cells to image (Figure S1A), 0.3% as the maximal tolerated DMSO concentration that does not affect speck formation (Figure S1B), 2 h LPS (1 µg/ml) (Figures S1C and D), 2 h nigericin (10 µM) (Figures S1E and F) stimulation resulting in maximal speck formation.

**Figure 1:**
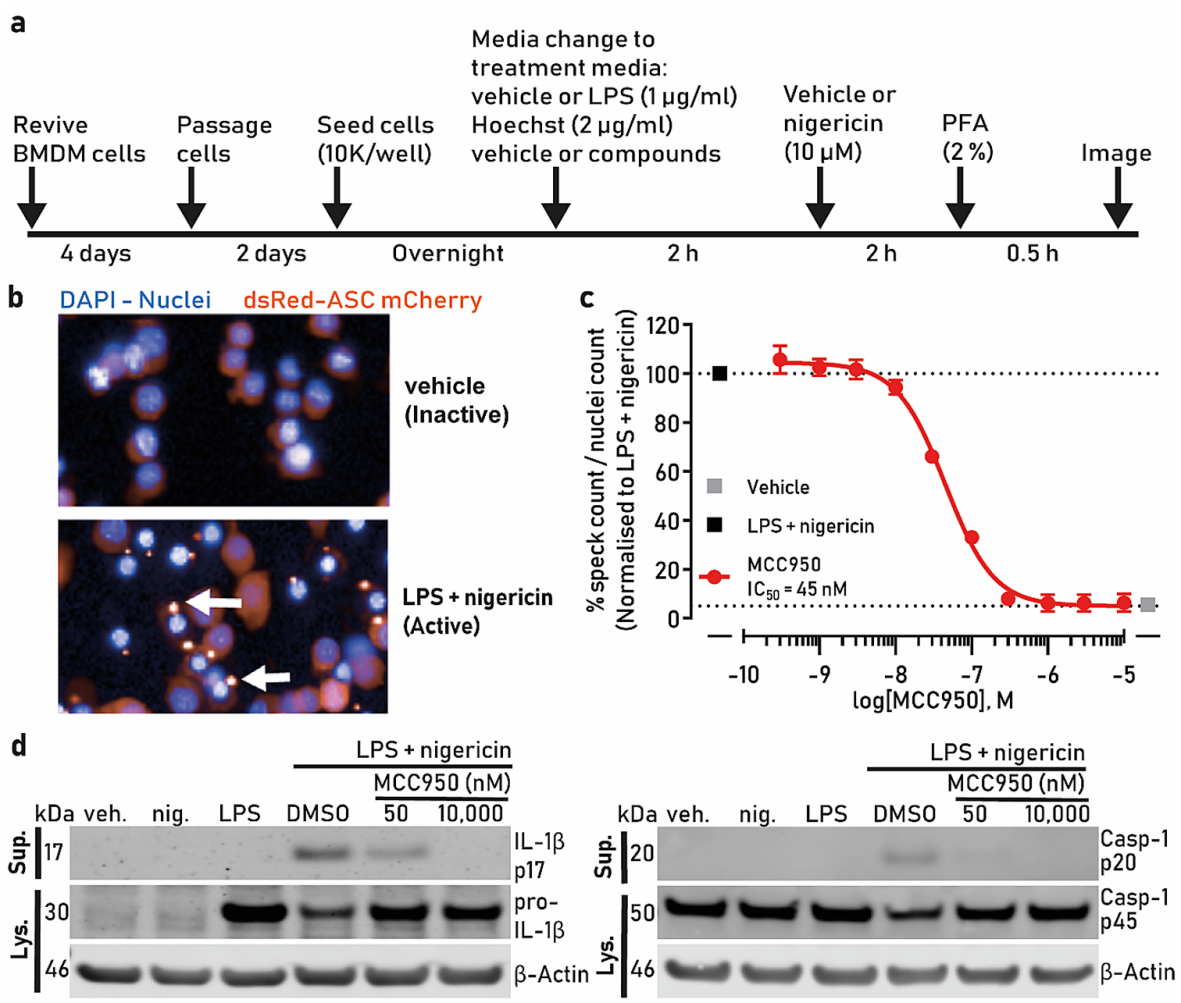
High-content imaging assay development. (**a**) Schematic of the assay protocol. (**b**) Untreated iBMDM cells show weak mCherry fluorescence, which strongly increases and localizes into a speck after inflammasome activation with LPS and nigericin (white arrows). Cell nuclei are visible in blue and ASC-mCherry in red. (**c**) ASC-mCherry iBMDM cells were treated with the indicated concentrations of MCC950 and LPS (1 µg/ml) for 2 h followed by nigericin treatment (10 µM, 2 h) after which PFA was added. The percentage of ASC specks/total nuclei normalised to LPS and nigericin is presented; (**d**) Immunoblot of pro-IL-1β and cleaved IL-1β (IL-1β p17), pro-caspase-1 (p45) and caspase-1 (p20) in the cell lysates and supernatants after cell stimulation with LPS + nigericin in the presence or absence of MCC950 (50 or 10,000 nM). β-actin was used as a loading control for the cell lysate samples. The IL-1β and caspase-1 bands are cropped from their respective blot images and the full-length blots are presented in Figure S5. Data in (**c**) are presented as mean ± SEM, n = 3 independent experiments and was analyzed using GraphPad Prism version 7 software (https://www.graphpad.com/scientific-software/prism/).

A summary schematic of the screening protocol is shown in Fig. 1a and the methodology is described in the screening assay section. Under basal conditions, the ASC-mCherry fluorescence remained weak and diffuse in the cytoplasm (Fig. 1b), while addition of LPS and nigericin resulted in an increase in fluorescence that concentrated into an intracellular speck in approximately 35–50% of cells, indicative of NLRP3 inflammasome activation (Fig. 1b arrows). We tested the well-described NLRP3 inhibitor MCC950 against nigericin-induced speck formation and obtained an IC_50_ of 45 nM (Fig. 1c), which is consistent with literature reports^16^. To further characterize MCC950’s effects on NLRP3 we immunoblotted the cell lysates for pro-IL-1β and pro-caspase-1 and the cell supernatants for mature IL-1β and mature caspase-1. We observed an increase in pro-IL1β expression after LPS treatment and an increase in the release of mature IL-1β and caspase-1 after nigericin addition. These effects were greatly reduced by MCC950 at 50 nM or 10 µM (Fig. 1d) corroborating the speck data, which are indicative of MCC950 inhibition of NLRP3 inflammasome.

**Figure 2:**
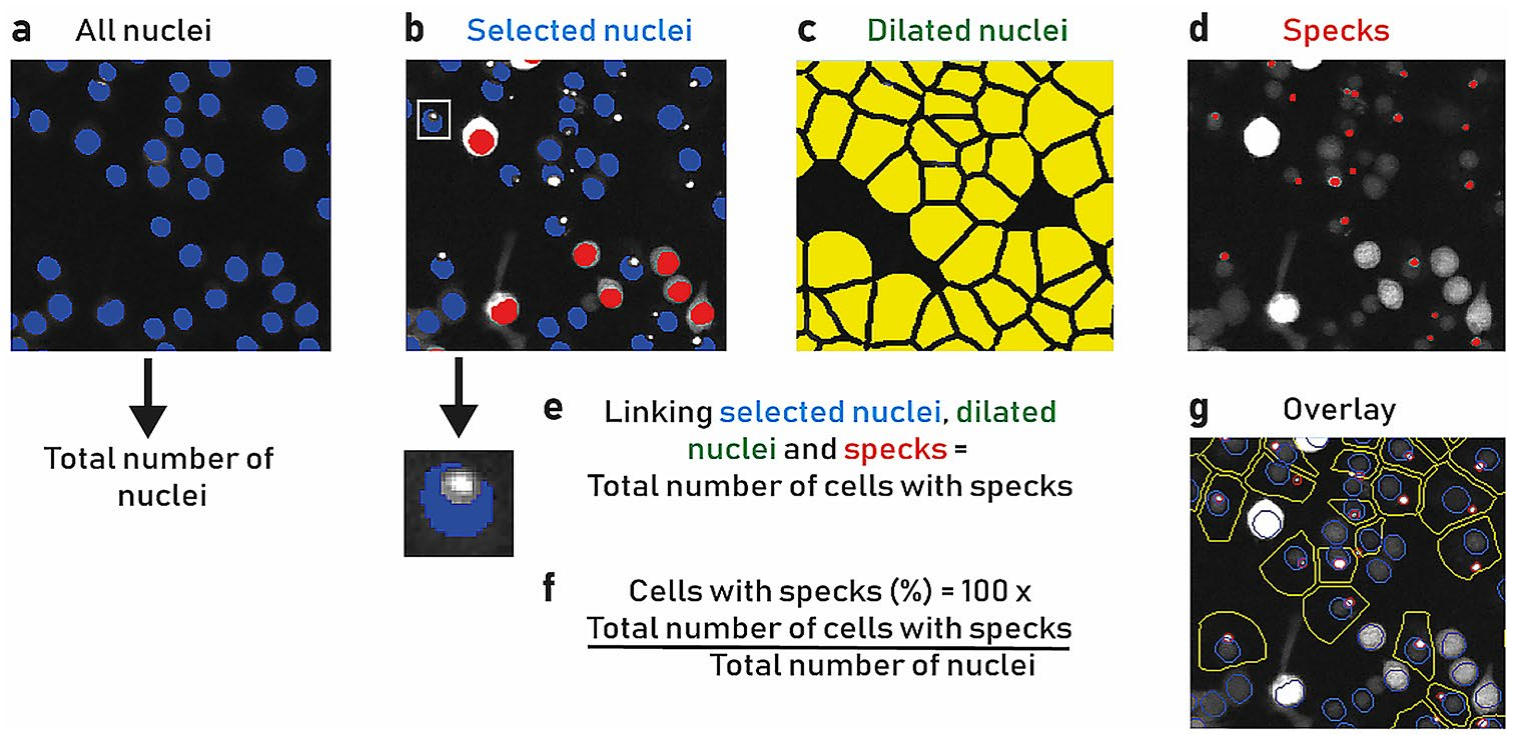
Speck image analysis pipeline. (**a**) All nuclei (blue) were segmented, and the total number of nuclei were counted. (**b**) Very bright mCherry fluorescent cells were removed from the analysis to avoid incorrect speck segmentation. This was achieved by measuring the mCherry intensity in the nucleus minus spot masks (see inset) and rejecting cells above a fixed intensity level threshold (red) and selected nuclei (blue) were retained. (**c**) Nuclei were dilated (yellow) to give a pseudo cell region where specks were counted. (**d**) Specks were segmented. (**e**) The selected nuclei, dilated nuclei and specks were linked together to yield the total number of cells with specks. (**f**) The final calculation consisted of the ratio between the total number of cells with specks and the total number of nuclei and multiplying it to 100 to calculate the percentage of cells with specks. (**g**) Example segmentation overlay after analysis, nuclei (blue), cells (yellow) and specks (red).

Image analysis protocol gave robust segmentation of the target regions and identification of cells with specks using a custom image analysis pipeline in Developer Toolbox 1.9.2 (GE Healthcare, Lifesciences). All nuclei were segmented, and the total number of nuclei counted (Fig. 2a). Over segmentation of non-specks in cells that were overly bright in the mCherry channel were filtered out from the analysis using an intensity-based threshold (Fig. 2b). Cell boundary was made to measure specks within the cells because mCherry-ASC moves from the cytoplasm to the NLRP3 complex. Therefore, the nucleus was dilated to give a pseudo cell region (Fig. 2c). The specks were subsequently segmented (Fig. 2d) and three main target regions; nuclei (selected nuclei), cell (dilated nuclei) and specks were linked together to give a new target set, a cell with a nucleus and at least one speck (Fig. 2e). The final calculation of the analysis is the percentage of cells with specks, which is conducted by dividing the total number of cells with specks by the total number of nuclei and multiplied by 100 (Fig. 2f). The result of the analysis is shown in an example of the segmentation with an overlay (Fig. 2g). Having established the optimal speck assay conditions including the use of a positive control, MCC950, we progressed to assay automation using liquid handlers. To confirm the liquid handling robotics method, we followed the NIH protocol^22^ and carried out inter- and intra-plate uniformity assessments using three 384-well plates over two separate days. Plate uniformity assessment checklist and assessment metrics (Table S1 and S2), both passed the measurement criteria. In the first robustness test, plates containing columns of +/− nigericin (max and min specks respectively) were prepared and evaluated. Assay metrics, averaged over the plates, demonstrated that the assay plates met acceptable cell-based assay quality control (QC) specifications (Z′ = 0.70 (Spec > 0.6); SSMD = 12 (Spec > 5); % CV maximum speck number = 9 (Spec < 10)). In the second robustness test, a dummy pilot of plates was conducted, using the same plate layout as a screen plate, dispensing DMSO into the 280 compound library positions and adding nigericin to all positions apart from cells alone and DMSO vehicle negative control wells. Assay metrics for the plates demonstrated that they met QC specifications (Z′ = 0.73 (Spec > 0.6); SSMD = 13.5 (Spec > 5); % CV maximum speck number = 8.3 (Spec < 10); % CV dummy library (DMSO) = 9.4). In both cases, the assay performed within specification, generated acceptable quality metrics and was amenable to automation.

### High-throughput screening

**Figure 3:**
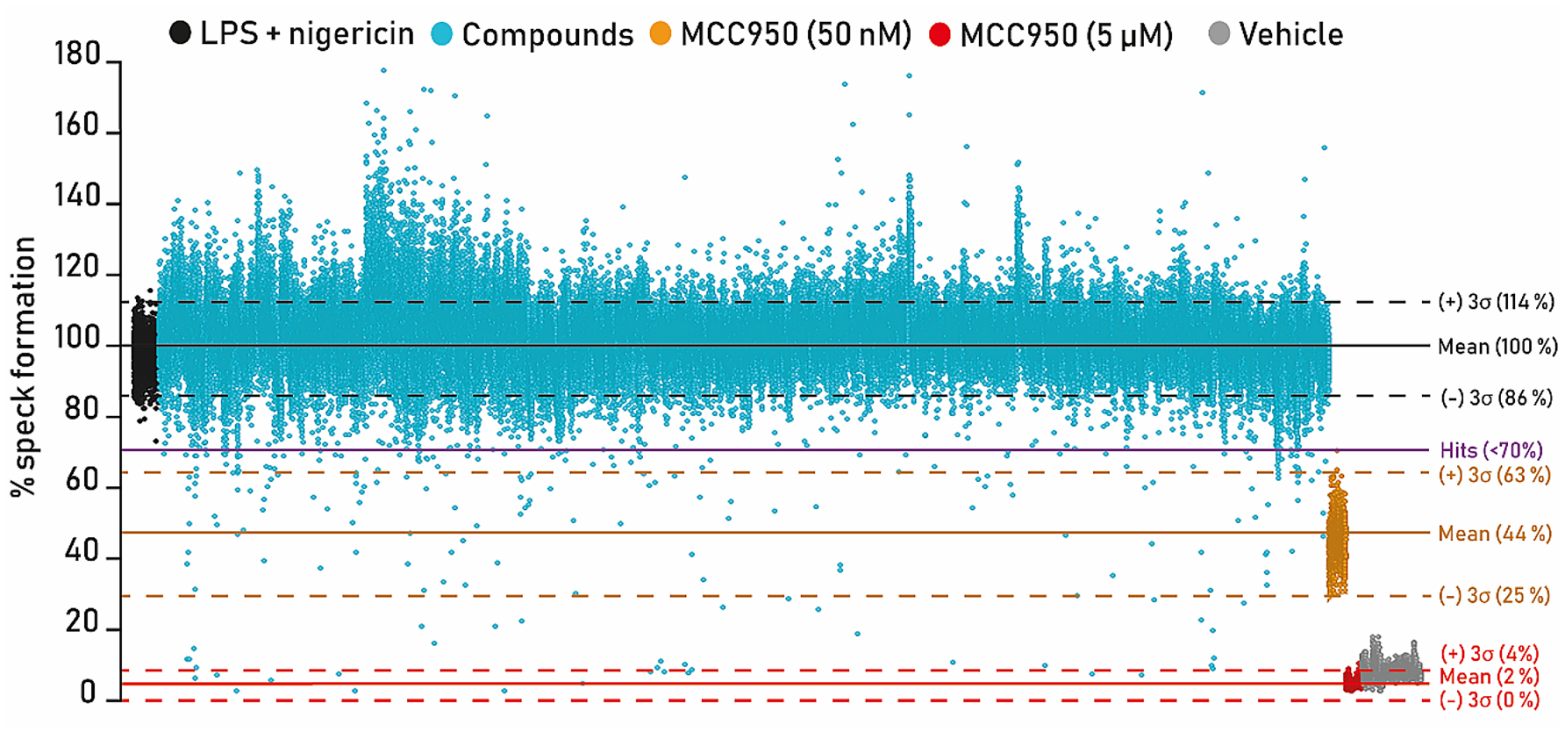
High-throughput screening, identification of hit compounds. ASC-mCherry iBMDM cells were treated with 5 µM of each of the 81,000 compounds (represented by each blue dot) and LPS (1 µg/ml) for 2 h followed by nigericin treatment (10 µM, 2 h) after which PFA was added. The percentage (%) speck formation of ASC specks/total nuclei normalised to LPS + nigericin are presented; LPS + nigericin treated wells are indicated in black on the left-hand side. MCC950 50 nM treated wells are indicated in orange (right hand side), while MCC950 5 µM treated wells are in red (on the right hand side), representing complete speck inhibition. Vehicle treated cells are on the right in grey. Data was analyzed using TIBCO Spotfire version 11 software (https://www.tibco.com/products/tibco-spotfire).

The JumpStarter and annotated libraries of 81,000 small molecule compounds were screened in the speck assay at the single concentration of 5 µM. MCC950 was used as the assay positive control at 50 nM (value close to IC_50_) and at 5 µM, a supramaximal NLRP3 inhibiting concentration. The first 10,000 compounds were run in duplicate with assay metrics demonstrating assay robustness (averaged data for all plates gave rZ′ 5 µM MCC950 = 0.82 (Spec > 0.6); rZ′ 50 nM MCC950 = 0.54 (Spec > 0.2); SSMD 5 µM MCC950 = 18.53 (Spec > 5); % CV maximum speck number = 6.12 (Spec < 10)). Comparing data from the two replicate plates, the Pearson correlation was = 0.93 (Spec > 0.9) average of all plate pairs. R1 vs R2 data were plotted in Spotfire Decision Site version 8.2 generating an r of 0.93 (Figure S2). To accelerate the primary screen while maximizing resources, we then conducted a single concentration primary screen on the remaining 71,000 compounds. Assay quality metrics for the single point primary screen were rZ′ = 0.86 for 5 µM MCC950, rZ′ = 0.62 for MCC950 50 nM, SSMD = 24 and CV = 5% for the negative control; all data was averaged across the 360 screening plates (Table 1) and plates which failed quality control metrics were repeated (5/347 plates failed, which is 1.4%). Compounds that inhibited speck formation by at least 30% scored as a positive hit. Using these criteria, we identified 244 hits with a hit rate of 0.3% (Fig. 3). Interestingly, several hits increased speck formation, which may be of interest mechanistically for therapeutic indications where inflammasome activation may prove beneficial. In this study, however, we were interested in identifying inhibitors of NLRP3 inflammasome activation, hence we took the inhibitory hits forward for validation.

**Table 1:** Speck assay 81,000 high-throughput screening metrics.

Metric	Specification (Spec)	Average (across all plates)	SD
Robust Z′ (positive control, MCC950 5 µM)	> 0.6	0.9	0.1
Robust Z′ (positive control, MCC950 50 nM = IC_50_)	> 0.2	0.6	0.2
SSMD	> 5	24	13.6
% CV (negative control, maximum speck number)	< 10	5	2.3

Assay plates were subjected to quality control analysis, evaluating the performance of each plate using positive (MCC950 50 nM and 5 µM) and negative (vehicle, DMSO) controls. The quality assay control acceptance criteria selected were robust Z′ > 0.6 (MCC950 5 µM; maximum speck number reduction), robust Z′ > 0.2 (MCC950 50 nM = IC_50_). SSMD > 5, % CV < 10% (negative control, maximum speck number). Screening metric averages and standard deviations across plates are shown.

### Validation of inhibitors of NLRP3 inflammasome activation

**Figure 4:**
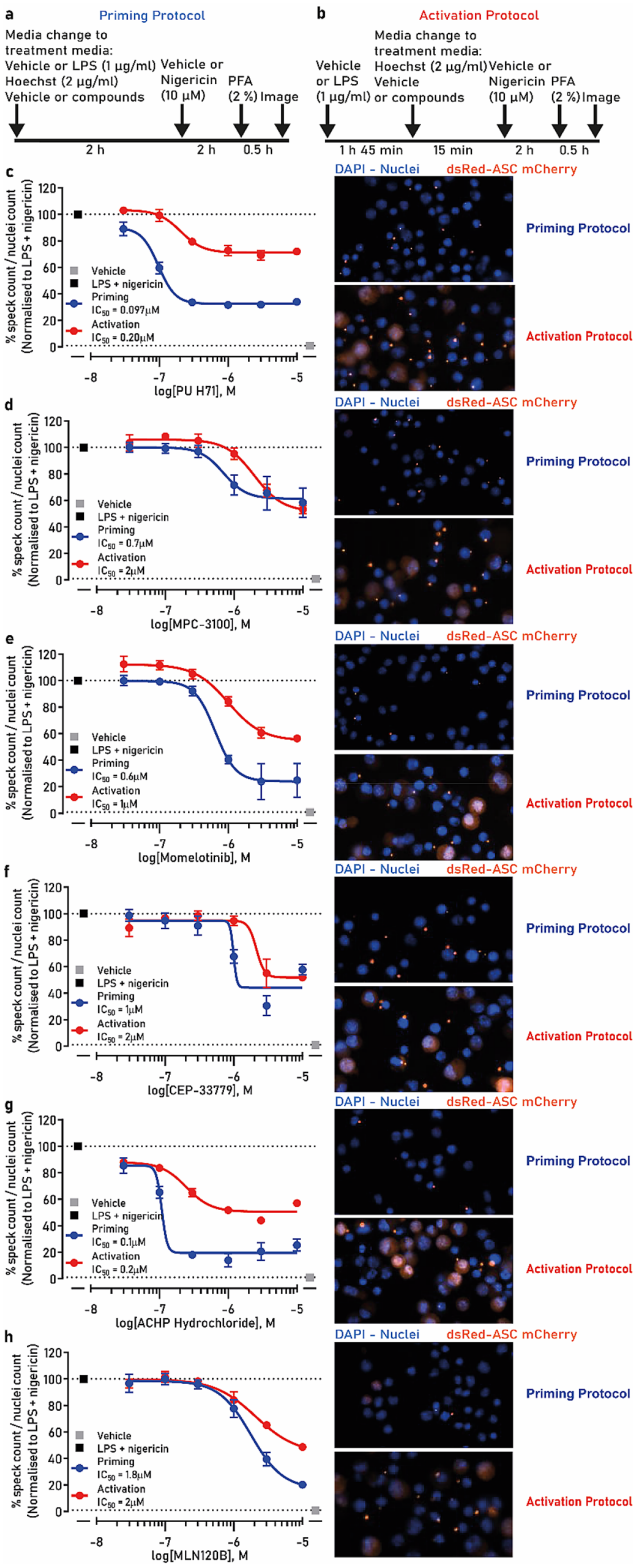
NLRP3 inflammasome inhibitor hits validation in the speck assay. (**a**) Schematic of the priming protocol. (**b**) Schematic of the activation protocol. ASC-mCherry iBMDM cells were treated with indicated concentrations of (**c**) PU H71, (**d**) MPC-3100, (**e**) momelotinib, (**f**) CEP-33779, (**g**) ACHP or (**h**) MLN120B with LPS (1 µg/ml) for 2 h followed by nigericin treatment (10 µM, 2 h) after which PFA was added (priming—blue trace) and specks counted. In a parallel experiment the compounds were added after LPS treatment just prior to nigericin (activation—red trace). The images are representative of the nuclei and speck formation for each compound at 10 µM in both priming and activation protocols. Data are presented as mean ± SEM, n = 3 independent experiments. Data in (**c**–**h**) was analyzed using GraphPad Prism version 7 software (https://www.graphpad.com/scientific-software/prism/).

From the hits that inhibited NLRP3-dependent ASC speck formation, we selected a sub-set with the existing protein target annotation for validation. Such compounds were inhibitors of: HSP90 (PU H71 and MPC-3100), JAK1,2 (momelotinib and CEP-33779) and IKK-β (ACHP and MLN120B). These compounds were chosen because they have been previously therapeutically evaluated and were retested in the speck assay to generate a relative IC_50_ (Fig. 4c–h). To better understand their mechanism of action, compounds were added at the time of LPS addition to assess their effect on priming (priming protocol, Fig. 4a) and to reveal any potential effect at the activation stage, compounds were added after LPS (activation protocol, Fig. 4b). We previously showed that the IC_50_ of our positive control MCC950 was the same in the priming and activation protocols indicating that MCC950 is involved in the activation stage of the NLRP3 inflammasome complex formation^23^.

PU H71 inhibited speck formation with an IC_50_ of 0.097 µM in the priming protocol and 0.2 µM in the activation protocol. The compound’s efficacy in reducing speck formation was incomplete reaching a maximal 60% (priming protocol) (Fig. 4c). The second HSP90 inhibitor we tested, MPC-3100, was less potent generating an IC_50_ of 0.7 µM and 2 µM in the priming versus activation protocols, respectively (Fig. 4d).

In contrast, the JAK inhibitor momelotinib inhibited speck formation, when used in the priming mode protocol, but was less potent than PU H71 with an IC_50_ of 0.6 µM. In the activation mode protocol, momelotinib showed even more reduced potency (IC_50_ of 1 µM) and partial speck reduction (50%) (Fig. 4e). CEP-33779 was also more potent when used in the priming mode, IC_50_ of 1 µM, compared with IC_50_ of 2 µM in the activation protocol with partial speck reduction in both cases (~ 50%) (Fig. 4f).

The IKK-β inhibitors ACHP and MLN120B almost completely inhibited speck formation when used in the priming mode protocol, with an IC_50_ of 0.1 µM and 1.8 µM, respectively. In the activation protocol, their efficacy, as well as potency, were marginally reduced (IC_50_ of 0.2 µM and 2 µM, for ACHP and MLN120B, respectively) (Fig. 4g, h).

These data validated the screening hits generating concentration–response curves and IC_50_ values, enabling comparison of compound potency and efficacy in the speck assay. Additionally, by utilizing the priming and activation protocols, we have gained insights on their mechanism of action. In all cases, HSP90, JAK and IKK-β inhibitors were demonstrated to be acting in the priming stage of NLRP3 inflammasome activation.

Interestingly, when the screening hits were analyzed in the priming versus activation mode paradigms, we were able to identify clusters of compounds. Multidimensional properties such as (MW, cLogP, pTSA, H-acceptors, H-donors, rotatable bonds, basic nitrogens and stereo centers) from each of the screened compounds were scored and then represented along the x and y axis. The compounds have been colored coded and split into three categories according to inhibition criteria A (green): compounds with maximum inhibition and standard sigmoidal curve, B (yellow): compounds that had maximum inhibitory effect but steep curve, and C (red): compounds with incomplete curves but with at least 30% inhibition. Moreover, three different shapes have been assigned to the compounds according to the activation mode they follow (primers are represented by dots, activators with a triangle and compounds behaving both as activators and primers with a rhombus). Interestingly three main clusters (x,y): (0,0.5);(0.5,0);(2,0.5) can be identified within the plot suggesting that compounds with similar multidimensional properties share the same mechanism of action (Figure S3).

### Compound effect on NLRP3 downstream assays (IL-1β, caspase-1, pyroptosis) and toxicity triaging

To establish and validate additional NLRP3 inflammasome readouts to enable compound progression through the screening cascade and to gain additional understanding of the hits’ mechanism of action we utilized western blotting for caspase-1 and IL-1β, Caspase-1 Glo assay, ELISA IL-1β, LDH cytotoxicity assay and CCK-8 kit toxicity assays and compared the effect of MCC950 with HSP90, JAK and IKK-β inhibitors.

Because the HSP90, JAK and IKK-β inhibitors worked better in the speck assay when the priming mode protocol was used, we continued to use this paradigm and collected cell supernatants after stimulation with LPS and nigericin.

**Figure 5:**
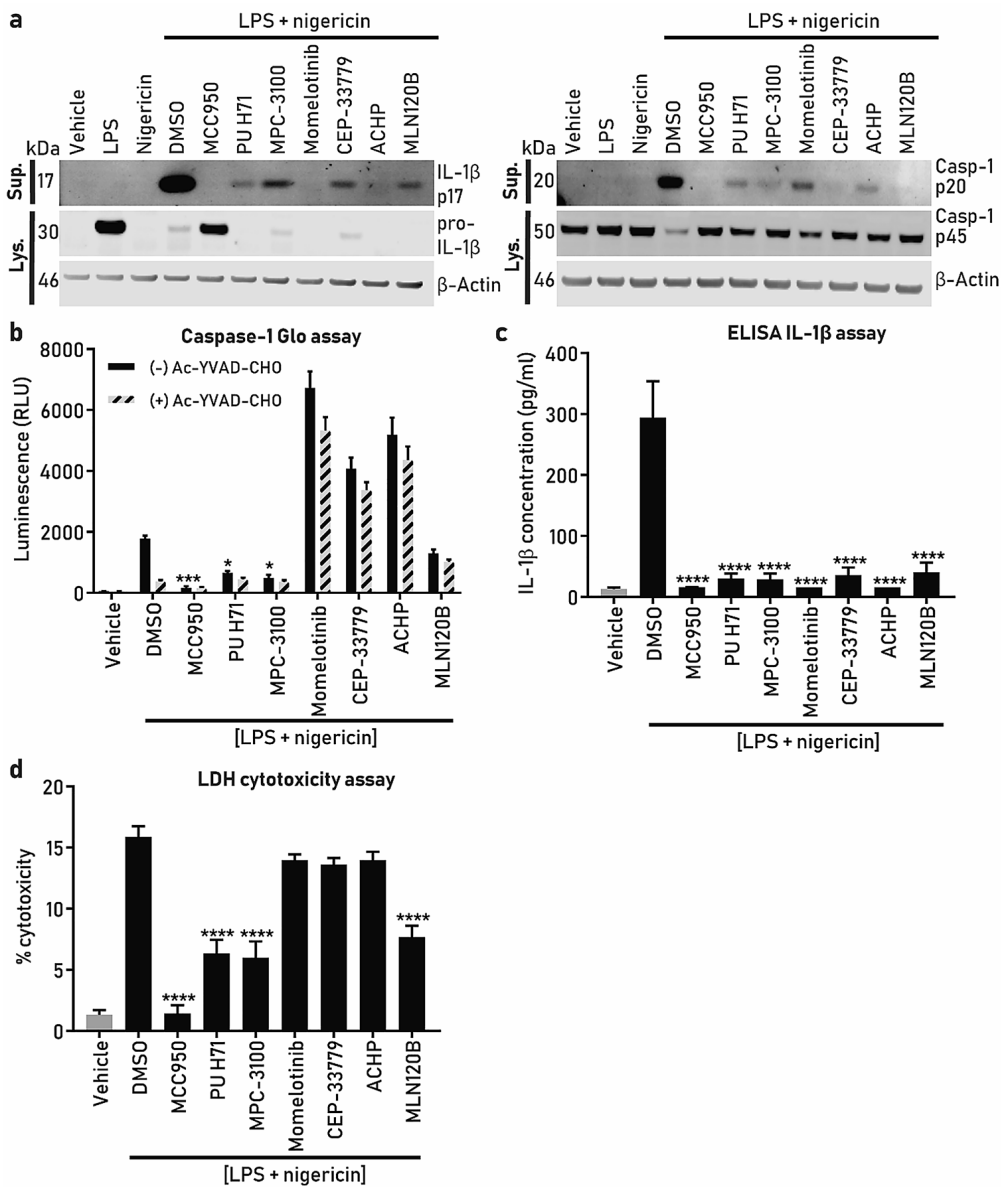
Compound effects on NLRP3 downstream assays (caspase-1, IL1-β, pyroptosis). ASC-mCherry iBMDM were treated with the indicated compounds (10 µM) at the same time as LPS (1 µg/ml, 2 h) and subsequently stimulated with nigericin (10 µM, 2 h). (**a**) Immunoblot of pro-IL-1β and cleaved IL-1β (IL-1β p17), pro-caspase-1 (p45) and caspase-1 (p20) in the cell lysates and supernatants after cell stimulation with LPS + nigericin in the presence or absence of compounds. β-actin was used as a loading control for the cell lysate samples. The IL-1β and caspase-1 bands are cropped from their respective blot images and the full-length blots are presented in Figure S6. (**b**) Caspase-1 activity using Caspase-1 Glo assay with the clear bars are (−) Ac-YVAD-CHO (caspase-1 inhibitor), while dashed lines bar are (+) Ac-YVAD-CHO, (**c**) IL-1β release using ELISA and (**d**) LDH release. In all experiments (**b**–**d**) data is presented as the mean ± SEM, n = 4 independent experiments and one-way ANOVA with post-hoc Dunnett’s test was utilized to calculate significance, *p < 0.05, **p < 0.01, ***p < 0.001, ****p < 0.0001. Data in (**b**–**d**) was analyzed using GraphPad Prism version 7 software (https://www.graphpad.com/scientific-software/prism/).

Western blotting of caspase-1 and IL-1β showed that the LPS + nigericin has a significant effect in releasing cleaved forms of caspase-1 and IL-1β and the pro forms are significantly decreased in the cell lysate (Fig. 5a). MCC950 is able to reverse these effects showing that these are driven through the NLRP3 inflammasome activation. All the other compounds examined showed inhibition of the cleaved IL-1β and caspase-1 p20 in the supernatants and interestingly, changes in pro-IL1β levels in the lysates. The HSP90 inhibitors PU H71 and MPC-3100 significantly inhibited caspase-1 activity, while the other tested compounds had a marginally reduced effect (MLN120B) or highly increased the signal, in the absence or presence of the caspase-1 inhibitor Ac-YVAD-CHO (Fig. 5b). Because Ac-YVAD-CHO was used to determine caspase-1 selectivity, the observed increase in luminescence with momelotinib, CEP-33779 and ACHP may result from activation of other caspases. Alternatively, the compounds may be interfering with the assay, so we tested them in the caspase-1 assay without LPS and nigericin stimulation. Interestingly, momelotinib and ACHP increased the caspase-1 luminescence signal when added to the cells without LPS and nigericin (Figure S4A).

Since caspase-1 cleaves pro-IL-1β to mature IL-1β we decided to measure IL-1β levels in the same cell supernatants. Interestingly, all the compounds tested were effective at completely inhibiting IL-1β release (Fig. 5c), corroborating the western blot data (Fig. 5a).

Additionally, because NLRP3 activation induces cell pyroptosis we measured LDH release, as a measure of cell death. In this assay, MCC950 was the most potent pyroptosis inhibitor, while the HSP90 inhibitors PU H71 and MPC-3100 and the IKK-β inhibitor MLN120B partially protected from cell death. Momelotinib, CEP-33779 and ACHP did not alter LDH release (Fig. 5d).

The results of the caspase-1, IL-1β and pyroptosis assays demonstrated compound differences reflecting differential mechanism of action of inflammasome regulation that would be interesting to explore further.

In order to determine if some of the effects observed with the compounds were related to cell toxicity, we treated iBMDMs with each of the compounds (in the absence of LPS and nigericin) for 4, 8 and 24 h and assessed cell viability using the cell counting kit-8.

After 4 h incubation, only ACHP and CEP-33779 showed 20% cytotoxicity at 10 µM (approximately 1–2 orders of magnitude higher than the priming and activation activity of ACHP and CEP-33779), however, all compounds, except MCC950, decreased cell viability at 8 and 24 h (Figure S4C, D, E). Interestingly, utilizing the LDH cytotoxicity assay only momelotinib and ACHP showed a significant increase in LDH release after 4 h incubation (Figure S4B).

Therefore, these data indicate that at the 4 h drug stimulation time point used in the NLRP3 inflammasome assays, the toxicity of ACHP, CEP-33779, and momelotinib could have negatively affected speck formation. Importantly, longer treatment results in cell toxicity therefore limiting the compounds use in in vitro assays.

## Discussion

The role of NLRP3 in the etiology or progression of several pathologies has generated intense interest and efforts to identify small molecule compound inhibitors of this inflammasome. MCC950 was originally identified from a phenotypic screening as an inhibitor of IL-1β production and only recently its molecular target has emerged as NLRP3 itself^11,12^. MCC950 has been shown to be effective in vitro and in a number of diseases in vivo rodent models^16,24,25^ decreasing inflammation, reducing pathology and protecting from cognitive decline^26,27^.

We therefore embarked into a screening campaign to identify novel inhibitors of NLRP3 activation as potential starting points for therapeutic development. A phenotypic screening approach was used to identify compounds with diverse mechanism of action in the NLRP3 pathway and to enable subsequent selection of those with a better safety profile for further development. We utilized an image-based NLRP3 speck formation assay, rather than an inflammasome downstream readout, such as IL-1β secretion or pyroptosis, to maximize our chances to identify selective inhibitors acting upstream in the pathway. This is important because there are NLRP3 inflammasome-independent pathways that modulate IL-1β processing, such as caspase-8 mediated conversion of pro-IL1β to mature IL-1β^28,29^. Additionally, we wanted to avoid compound assay interference that could emerge from using fluorimetric or luminescence-based assays such as caspase-1 or pyroptosis. Imaging flow cytometry to detect ASC specks in human monocytes has been previously utilized^30^. However, the method described in the paper did not result in the identification of useful chemical compounds and in fact MCC950 did not work in the assay, raising the question of how much the readout was NLRP3 mediated. Interestingly, an image-based ASC speck assay using immortalized human THP-1 macrophages was described by Redondo-Castro et al.^31^, demonstrating potential for utilization in screening mode as the assay was established in a 96-well plate format. However, the assay was utilized for purposes other than high-throughput screening, rather to identify a toolbox of NLRP3 inhibitors.

We capitalized on the assay initially described by Daniels et al.^21^ utilizing iBMDM cells stably transfected with a fluorescent tag on the ASC protein^32^. Although relying on a mouse cell background, selection of this cell line allowed assay simplification as it did not require the additional step of differentiation into macrophages, which impacts screening timelines, cost and potentially increases variability. Additionally, this cell line has a short doubling time allowing quick stock re-supply, beneficial for high-throughput screening. In basal conditions the mCherry fluorescence appears weak and diffuse in the cytosol, however, after treatment with LPS and nigericin, fluorescence nicely concentrated into an intracellular bright speck of approximately 1 µm size, which is easily and accurately quantified using the platform that we have utilized and optimized. We were fortunate to have a positive control in the screening assay, MCC950, which allowed robust metrics generation. Z′ factor, CV, signal window and signal-to-background ratio reflected the robustness of the speck assay as it performed consistently over the course of the screen of 360 plates (Table 1). Several positive hit molecules were identified from the 81,000 small molecule compound library, indicating the potential of the assay to identify novel chemical starting points and study mechanisms of speck regulation. Interestingly, a number of hits were identified that increased speck formation compared with LPS/nigericin treatment, which may be of interest for mechanistic NLRP3 studies, as well as for therapeutic opportunities in certain diseases such as colitis-associated colorectal cancer^33^, where activation of the NLRP3 inflammasome may be beneficial. However, as our interest lies in neurodegeneration, we followed up a number of speck inhibitor hits and reconfirmed their effect on speck formation in more detail by generating IC_50_ data.

The hits that we selected were target-annotated allowing us to gain insights on HSP90, JAK and IKK-β regulation of the NLRP3 pathway utilizing NLRP3-downstream cellular readouts, such as caspase-1, IL-1β and pyroptosis. Interestingly, these targets have been reported in the literature to regulate the inflammasome. HSP90 has been shown to bind NLRP3, regulate its stability and modulate inflammasome activation including IL-1β secretion and pyroptosis^23,34–37^. The JAK inhibitor tofacitinib was reported to inhibit NLRP3 inflammasome induced by granulocyte–macrophage colony-stimulating factor^38^. Inhibition of IKK-β has been shown to prevent NF-кB translocation subsequently decreasing NLRP3 expression^39^.

We have studied the compound mechanism of speck inhibition by testing the compounds using the priming, as well as activation mode protocols, revealing a more pronounced effect when added at the time of LPS addition, therefore indicating to primarily inhibit NLRP3 priming, by decreasing NLRP3 expression, modulating post-translational modifications or both. We also revealed a partial effect on speck reduction, in contrast with MCC950 which completely inhibited speck formation. We speculate that it may be beneficial to partially inhibit NLRP3 inflammasome to prevent infection susceptibility, which could be a side effect of inflammasome inhibition. Interestingly, the effect of HSP90, JAK and IKK-β inhibitors on caspase-1, IL-1β and pyroptosis showed dissimilar effects in the assays, with all tested compounds equally blocking IL-1β release, but only the HSP90 inhibitors also inhibiting pyroptosis. The differential effects of the compounds on inflammasome-mediated readouts may be related to their different mechanism of action on inflammasome regulation, which will be interesting to explore in future studies. Compound interference with the caspase-1 assay was observed in some cases. Additionally, while at the 4 h time point utilized in the inflammasome assays marginal cell toxicity was observed, it is important to note that longer time treatment (8 and 24 h) resulted in pronounced in vitro toxicity that would have to be taken into consideration when progressing these compounds further. It is interesting to note that MCC950 inhibited speck formation, as well as caspase-1, IL-1β and protected from pyroptosis and, in addition, it demonstrated a good safety profile in cells.

Further, to overcome potential safety issues in the clinic, we suggest NLRP3 inhibitors should be selective versus the other inflammasomes, such as NLRC4 and AIM2, to avoid the risk of compromising the immune system and allow it to react to PAMPs and DAMPs. Therefore, it would be beneficial to incorporate such selectivity assays in the screening cascade.

Lastly, it would be interesting to test the most selective and potent NLRP3 inhibitors in several in vivo disease models, including neurodegeneration to evaluate their therapeutic potential.

## Methods

### Materials and reagents

ASC-mCherry stably transfected iBMDM cells were described previously^21^. Lipopolysaccharides from Escherichia coli O55:B5 (LPS) (Cat. number: L6529), phosphate-buffered saline (PBS) (Cat. number: D8662), penicillin/streptomycin (CAS number: 93572-42-0), dimethyl sulphoxide (DMSO) (CAS number: 67-68-5), nigericin (CAS number: 64-17-5), PhosSTOP (Cat. number: 4906837001), Tween 20 (Cat. number: P1379), cell counting kit 8 (Cat. number: 64987), bovine serum albumin (BSA) (Cat. number: A7906), LDH activity assay kit (Cat. number: MAK066) and cell counting kit-8 (CCK-8) (Cat. number: 96992) were purchased from Merck; paraformaldehyde (PFA) (CAS number: 30525-89-4) and Tris buffered saline (TBS) with Tween 20 (Cat. number: sc-24953) were purchased from Santa Cruz; DMEM cell culture media (Cat. number: 31966021), DMEM for treatment media (Cat. number: 21063029), fetal bovine serum (FBS) (Cat. number: 100500064), TrypLE (Cat. number: 12605010), Hoechst 33,342 (Cat. number: H3570), RIPA buffer (Cat. number: 89900), NuPAGE sample reducing agent (Cat. number: NP0004) and PageRuler (Cat. number: 26619) were purchased from ThermoFisher Scientific; anti-caspase-1(p20) antibody (Cat. number: AG-20B-0042) was purchased from Adipogen; anti-IL-1β /IL-1F2 antibody (Cat. number: AF-401-NA) and mouse IL-1β/IL-1F2 DuoSet ELISA (Cat. number: DY401) were purchased from R&D Systems; rabbit anti-β-actin (Cat. number: 4970) and mouse anti-β-actin (Cat. number: 3700) antibodies were purchased from Cell Signaling Technology. Caspase-Glo 1 Inflammasome Assay (Cat. number: G9951) was purchased from Promega.

CellCarrier 384 black plates (Cat. number: 6007550) and P30 tips MDT sterile clear RoboRack MDT (Cat. number: 6001287) were purchased from Perkin Elmer; storage plates 384-well round bottom (Cat. number: 3656) were purchased from Costar; ECHO qualified 384-well, polypropylene microplates, clear flat bottom (Cat. number: P-05525) were purchased from Labcyte.

MCC950 (CAS number: 256373-96-3) was purchased from Tocris; PU H71 (Cat. number: 1856) and momelotinib (CAS number: 1056634-68-4) were purchased from Axon Medchem; MPC-3100 (Cat. number: A4063) was purchased from ApexBio; MLN120B (CAS number: 783348-36-7), CEP-33779 (CAS number: 958025-66-6) and ACHP (CAS number: 406209-26-5) were purchased from MedChemExpress.

The small molecule compound library was kindly provided by Janssen Pharmaceuticals via the Phenomics Discovery initiative. The library comprised two collections, 80,000 compounds in the ‘JumpStarter’ library suitable for hit identification and chosen to be diverse, lead-like (i.e., > 95% of compounds with MW < 450, alogP < 4.5) and free from contractual limitations^40^. The second ‘annotated’ set was selected to assist phenotypic screening. It consisted of 1000 advanced Janssen leads or reported tool compounds with primary bioactivity < 100 nM (67% with < 10 nM) across a range of drug-targets (844 target proteins) and with many cases including selectivity assessed by broad screening.

### Cell culture

ASC-mCherry expressing iBMDMs were grown in culture media made of DMEM, 10% (v/v) FBS, 1% (v/v) penicillin/streptomycin at 37 °C/5% CO_2_. iBMDM cells were trypsinized with TrypLE for 5 min and centrifuged at 1000×*g.* Cell count and viability were measured using a cell counter and cells seeded for the different experiments as described in the relevant sections. Before starting the screening, cells were expanded and stored in liquid nitrogen in individual cryovials containing 5 million cells/ml. A total of 60 vials were used in the screen, using the same cell passage number for every screening batch.

### Western blotting

iBMDMs were seeded at 4 million cells/ml of culture media in 6-well plates. On the following day the media was replaced with treatment media with the addition of LPS (1 µg/ml) for 2 h followed by nigericin (10 µM) for 2 h. Supernatant and cell samples were lysed with RIPA buffer containing protease and phosphatase inhibitors and mixed with sample buffer containing reducing agent and denatured at 70 °C for 10 min. Samples (20 µg) and protein ladder were loaded onto 4–12% gradient Bis–Tris gels and separated at 200 V for 30 min. Proteins were transferred onto a nitrocellulose membrane at 100 V for 30 min. Membranes were blocked in TBS containing 5% (w/v) BSA with 0.1% (v/v) Tween 20 (blocking buffer) for 1 h, and incubated overnight with mouse anti-caspase-1 (1:1000) and rabbit anti-β-actin (1:1000) antibodies or rabbit anti-IL-1β (0.25 µg/ml) and mouse anti-β-actin (1:1000) antibodies in blocking buffer at 4 °C with gentle shaking. The membranes were washed three times with TBS containing 0.1% (v/v) Tween 20 and incubated with secondary antibodies LI-COR IRDye donkey anti-rabbit 800 (1:20,000) and LI-COR IRDye donkey anti-mouse (1:20,000) for 1 h at room temperature, washed 3 times as described above and then scanned simultaneously at 700 and 800 nm with the LI-COR Odyssey Imaging System.

### Screening assay

iBMDM cells were removed from the liquid nitrogen storage, a fresh vial for each batch of screening and revived in a T75 flask in culture media at 37 °C/5% CO_2_ (day = 1) (Fig. 1a). Cells were passaged in T175 flasks when confluent (day = 4) and grown for an additional 2 days before seeding them into 384-well plates at 10,000 cells/85 µl of culture media/well using a Flex Drop (Perkin Elmer, Waltham, MA, USA) bulk liquid handler (day = 6). Compound library plates were provided in ECHO LDV 384-well format and compounds were dispensed using the ECHO 550 (Labcyte, San Jose, CA, USA) such that a final concentration of 5 µM was achieved in the assay. For the screening assay, after overnight incubation (day = 7), the Perkin Elmer Janus automated liquid handler (Perkin Elmer, Waltham, MA, USA) was utilized to replace the culture media with treatment media containing LPS (1 µg/ml), Hoechst (2 µg/ml) and compounds. Plates were placed in an incubator at 37 °C/5% CO_2_ for 2 h. Nigericin (10 µM) was then added, and the plates further incubated at 37 °C/5% CO_2_ for 2 h. Because iBMDMs are weakly adherent cells, to retain the cells throughout the assay, no wash steps were conducted. Key to success of the liquid handling was the height of tip dispense (2 mm from the well bottom), speed of tip dispensing (1 µl/s), placing the plates in the incubator in a monolayer to ensure all plates reached the temperature and 5% CO_2_ in the 2 h incubation window. The experiment was terminated by adding PFA (final 2%) to fix the cells without any wash steps to avoid cell loss and after 30 min the plates were imaged.

In the 2 h time window between reagent additions we were able to process 16 × 384-well assay plates, equating to 4,480 compounds per run.

In the activation mode experiments, iBMDMs were stimulated with LPS (1 µg/ml) and placed at 37 °C/5% CO_2_ for 2 h. The culture media was then replaced with treatment media containing Hoechst (2 µg/ml) and compounds and incubated for 15 min. Nigericin (10 µM) was then added and plates further incubated for 2 h at 37 °C/5% CO_2_ before fixation, as described above.

### High-content imaging and analysis

Plates were imaged utilizing the GE IN Cell Analyzer 6000 (GE, Boston, MA, USA), 10x/0.45 Plan Apo CFI/60 objective, 2 fields of view, and two wavelengths/channels: dsRed (excitation: 561 nm, emission: 605 nm) to image mCherry specks and DAPI (excitation: 405 nm, emission: 455 nm) to image Hoechst stained nuclei.

Image analysis was performed using a custom protocol in IN Cell Developer Toolbox 1.9.2 software, enabling automated imaging then analysis during the screening process. The main analysis protocol steps were segmentation of the nuclei using the DAPI channel, segmentation of the ASC specks using the DsRed channel, dilation of the nuclear mask to generate a pseudo cell region, then target linking of ASC specks within this cell region (Fig. 2). The total number of cells and the number of cells with ASC specks were measured; from this, the percentage of cells with specks was calculated. In a protocol refinement step, cells with bright uniform mCherry signal were rejected before the target linking step to minimize the quantification of non-specks segmented by the error in these bright cells.

### Speck assay quality control and data analysis

All speck assay plates were subjected to quality control analysis, evaluating the performance of each plate using positive (MCC950 50 nM and 5 µM) and negative (vehicle, DMSO) controls. The quality metrics acceptance criteria selected were strictly standardized mean difference (SSMD) > 5, % coefficient of variation (CV) < 10% (negative controls: maximum speck number), robust Z′ > 0.6 (MCC950 5 µM: maximum speck number reduction), robust Z′ > 0.2 (MCC950 50 nM = IC_50_) (Table 1)^41^. All data were plotted in TIBCO Spotfire version 11 (https://www.tibco.com/products/tibco-spotfire) and GraphPad Prism version 7 (https://www.graphpad.com/scientific-software/prism/). Compounds were classified as positive hits if the percentage of speck inhibition was greater than 30%. Selected hits were validated by performing six-point, semi-log concentration response experiments and results generated using logistic regression curve fit (max = 100), four-parameter logistic model.

### NLRP3 screening cascade cellular assays (IL-1β, caspase-1, pyroptosis, toxicity triaging)

iBMDM cells were seeded at 400,000 cells/0.5 ml culture media/well in 24-well plates. The following day, the culture media was replaced with treatment media containing LPS (1 µg/ml) and compounds and placed at 37 °C/5% CO_2_ for 2 h. Nigericin (10 µM) was subsequently added and incubated in a 37 °C/5% CO_2_ for 2 h. The supernatants were harvested for the quantification of IL-1β by ELISA, cell death by LDH release, caspase-1 activity using a Caspase-1 GLO assay. A cytotoxicity assay was also carried out in which different concentrations of compounds were incubated with iBMDM cells over 4, 8 or 24 h and the CCK-8 kit was used to measure cell viability. The PHERAstar FSX plate reader was used to measure absorbance/luminescence. The assays were carried out following manufacturer’s instructions.

Data were analysed using GraphPad Prism version 7 software. Mean ± SEM were calculated, as indicated. Non-linear regression fit was used to generate the IC_50_. One-way ANOVA with post-hoc Dunnett’s test was utilised to calculate significance. In all experiments *p < 0.05, **p < 0.01, ***p < 0.001, ****p < 0.0001.

## Supplementary Information

Supplementary Information.

## References

[1] Guo H, Callaway JB, Ting JP (2015). Inflammasomes: Mechanism of action, role in disease, and therapeutics. Nat. Med..

[2] de Rivero Vaccari JP, Dietrich WD, Keane RW (2014). Activation and regulation of cellular inflammasomes: Gaps in our knowledge for central nervous system injury. J. Cereb. Blood Flow Metab..

[3] Walsh JG, Muruve DA, Power C (2014). Inflammasomes in the CNS. Nat. Rev. Neurosci..

[4] Place DE, Kanneganti TD (2018). Recent advances in inflammasome biology. Curr. Opin. Immunol..

[5] Schmid-Burgk JL (2016). A genome-wide CRISPR (clustered regularly interspaced short palindromic repeats) screen identifies NEK7 as an essential component of NLRP3 inflammasome activation. J. Biol. Chem..

[6] He Y, Zeng MY, Yang D, Motro B, Nunez G (2016). NEK7 is an essential mediator of NLRP3 activation downstream of potassium efflux. Nature.

[7] Shi H (2016). NLRP3 activation and mitosis are mutually exclusive events coordinated by NEK7, a new inflammasome component. Nat. Immunol..

[8] Nizami S, Hall-Roberts H, Warrier S, Cowley SA, Di Daniel E (2019). Microglial inflammation and phagocytosis in Alzheimer's disease: Potential therapeutic targets. Br. J. Pharmacol..

[9] Malik A, Kanneganti TD (2017). Inflammasome activation and assembly at a glance. J. Cell Sci..

[10] Shi J (2015). Cleavage of GSDMD by inflammatory caspases determines pyroptotic cell death. Nature.

[11] Tapia-Abellan A (2019). MCC950 closes the active conformation of NLRP3 to an inactive state. Nat. Chem. Biol..

[12] Coll RC (2019). MCC950 directly targets the NLRP3 ATP-hydrolysis motif for inflammasome inhibition. Nat. Chem. Biol..

[13] Cordero MD, Alcocer-Gomez E, Ryffel B (2018). Gain of function mutation and inflammasome driven diseases in human and mouse models. J. Autoimmun..

[14] Dinarello CA, van der Meer JW (2013). Treating inflammation by blocking interleukin-1 in humans. Semin. Immunol..

[15] Khan N, Kuo A, Brockman DA, Cooper MA, Smith MT (2018). Pharmacological inhibition of the NLRP3 inflammasome as a potential target for multiple sclerosis induced central neuropathic pain. Inflammopharmacology.

[16] Coll RC (2015). A small-molecule inhibitor of the NLRP3 inflammasome for the treatment of inflammatory diseases. Nat. Med..

[17] Pinkerton JW (2017). Inflammasomes in the lung. Mol. Immunol..

[18] Gordon R (2018). Inflammasome inhibition prevents α-synuclein pathology and dopaminergic neurodegeneration in mice. Sci. Transl. Med..

[19] Marín-Aguilar F (2020). NLRP3 inflammasome inhibition by MCC950 in aged mice improves health via enhanced autophagy and PPARα Activity. J. Gerontol. A Biol. Sci. Med. Sci..

[20] Mangan MSJ (2018). Targeting the NLRP3 inflammasome in inflammatory diseases. Nat. Rev. Drug Discov..

[21] Daniels MJ (2016). Fenamate NSAIDs inhibit the NLRP3 inflammasome and protect against Alzheimer's disease in rodent models. Nat. Commun..

[22] Iversen, P. W. *et al.* in *Assay Guidance Manual* (eds S. Markossian *et al.*) (Eli Lilly & Company and the National Center for Advancing Translational Sciences, 2004). 22553861

[23] Nizami S (2020). Inhibition of the NLRP3 inflammasome by HSP90 inhibitors. Immunology.

[24] Heneka MT (2013). NLRP3 is activated in Alzheimer's disease and contributes to pathology in APP/PS1 mice. Nature.

[25] Venegas C (2017). Microglia-derived ASC specks cross-seed amyloid-beta in Alzheimer's disease. Nature.

[26] Dempsey C (2017). Inhibiting the NLRP3 inflammasome with MCC950 promotes non-phlogistic clearance of amyloid-beta and cognitive function in APP/PS1 mice. Brain Behav. Immun..

[27] Ising C (2019). NLRP3 inflammasome activation drives tau pathology. Nature.

[28] Gringhuis SI (2012). Dectin-1 is an extracellular pathogen sensor for the induction and processing of IL-1beta via a noncanonical caspase-8 inflammasome. Nat. Immunol..

[29] Netea MG, van de Veerdonk FL, van der Meer JW, Dinarello CA, Joosten LA (2015). Inflammasome-independent regulation of IL-1-family cytokines. Annu. Rev. Immunol..

[30] Lage SL, Dominical VM, Wong CS, Sereti I (2019). Evaluation of canonical inflammasome activation in human monocytes by imaging flow cytometry. Front. Immunol..

[31] Redondo-Castro E (2018). Development of a characterised tool kit for the interrogation of NLRP3 inflammasome-dependent responses. Sci. Rep..

[32] De Nardo D, De Nardo CM, Latz E (2014). New insights into mechanisms controlling the NLRP3 inflammasome and its role in lung disease. Am. J. Pathol..

[33] Karki R, Man SM, Kanneganti TD (2017). Inflammasomes and cancer. Cancer Immunol. Res..

[34] Piippo N (2018). Hsp90 inhibition as a means to inhibit activation of the NLRP3 inflammasome. Sci. Rep..

[35] Mayor A, Martinon F, De Smedt T, Petrilli V, Tschopp J (2007). A crucial function of SGT1 and HSP90 in inflammasome activity links mammalian and plant innate immune responses. Nat. Immunol..

[36] Zhang M (2020). A translocation pathway for vesicle-mediated unconventional protein secretion. Cell.

[37] Zhou Z (2020). Heat shock protein 90 inhibitors suppress pyroptosis in THP-1 cells. Biochem. J..

[38] Furuya MY (2018). Tofacitinib inhibits granulocyte-macrophage colony-stimulating factor-induced NLRP3 inflammasome activation in human neutrophils. Arthritis Res. Ther..

[39] Irrera N (2017). BAY 11–7082 inhibits the NF-kappaB and NLRP3 inflammasome pathways and protects against IMQ-induced psoriasis. Clin. Sci. (Lond.).

[40] Hann MM, Oprea TI (2004). Pursuing the leadlikeness concept in pharmaceutical research. Curr. Opin. Chem. Biol..

[41] Zhang XD (2007). A pair of new statistical parameters for quality control in RNA interference high-throughput screening assays. Genomics.

