# Author Correction: A phenotypic high-content, high-throughput screen identifies inhibitors of NLRP3 inflammasome activation

**DOI:** 10.1038/s41598-021-00202-z

**Published:** 2021-10-11

**Authors:** Sohaib Nizami, Val Millar, Kanisa Arunasalam, Tryfon Zarganes-Tzitzikas, David Brough, Gary Tresadern, Paul E. Brennan, John B. Davis, Daniel Ebner, Elena Di Daniel

**Affiliations:** 1 Alzheimer’s Research UK Oxford Drug Discovery Institute, NDM Research Building, University of Oxford, Old Road Campus, Roosevelt Drive, Oxford, OX3 7FZ UK; 2 Target Discovery Institute, NDM Research Building, University of Oxford, Old Road Campus, Roosevelt Drive, Oxford, OX3 7FZ UK; 3 Division of Neuroscience and Experimental Psychology, School of Biological Sciences, Faculty of Biology, Medicine and Health, Manchester Academic Health Science Centre, University of Manchester, AV Hill Building, Oxford Road, Manchester, M13 9PT UK; 4 Lydia Becker Institute of Immunology and Inflammation, University of Manchester, Manchester, M13 9PT UK; 5 National Phenotypic Screening Centre, Target Discovery Institute, NDM Research Building, University of Oxford, Old Road Campus, Roosevelt Drive, Oxford, OX3 7FZ UK; 6 Janssen Research and Development, Turnhoutseweg 30, 2340 Beerse, Belgium

Correction to: *Scientific Reports* https://doi.org/10.1038/s41598-021-94850-w, published online 28 July 2021.

In the original version of this Article Sohaib Nizami and Val Millar were omitted as equally contributing authors. The original Article and accompanying Supplementary Information file have been corrected.

