# Supplementary material for: A phenotypic high-content, high-throughput screen identifies inhibitors of NLRP3 inflammasome activation
Source: Sci Rep. 2021 Jul 28;11:15319. doi: 10.1038/s41598-021-94850-w (PMC8319173; doi:10.1038/s41598-021-94850-w)
Supplement: Supplementary file 1 — Supplementary Information. [file 41598_2021_94850_MOESM1_ESM.docx]

# A phenotypic high-content, high-throughput screen identifies inhibitors of NLRP3 inflammasome activation

Sohaib Nizami^1,2^, Val Millar^1,2,5^, Kanisa Arunasalam^1,2^, Tryfon Zarganes-Tzitzikas^1,2^, David Brough^3,4^, Gary Tresadern^6^, Paul E. Brennan^1,2^, John B. Davis^1,2^, Daniel Ebner^2,5^* & Elena Di Daniel^1,2^*

^1^Alzheimer's Research UK Oxford Drug Discovery Institute, NDM Research Building,

University of Oxford, Old Road Campus, Roosevelt Drive, Oxford OX3 7FZ, UK.

^2^Target Discovery Institute, NDM Research Building, University of Oxford, Old Road Campus, Roosevelt Drive, Oxford OX3 7FZ, UK.

^3^Division of Neuroscience and Experimental Psychology, School of Biological Sciences, Faculty of Biology, Medicine and Health, Manchester Academic Health Science Centre, University of Manchester, AV Hill Building, Oxford Road, Manchester M13 9PT, UK.

^4^Lydia Becker Institute of Immunology and Inflammation, University of Manchester, Manchester M13 9PT, UK.

^5^National Phenotypic Screening Centre, Target Discovery Institute, NDM Research Building, University of Oxford, Old Road Campus, Roosevelt Drive, Oxford OX3 7FZ, UK.

^6^Janssen Research & Development, Turnhoutseweg 30, Beerse, B-2340, Belgium.

These authors contributed equally: Sohaib Nizami and Val Millar

*Corresponding authors:

Elena Di Daniel, Alzheimer's Research UK Oxford Drug Discovery Institute, NDM Research Building, University of Oxford, Old Road Campus, Roosevelt Drive, Oxford OX3 7FZ, email: elena.di-daniel@astx.com, phone: +44 1865 612 890, ORCID 0000-0002-0535-2123

Daniel Ebner, Target Discovery Institute, NDM Research Building, University of Oxford, Old Road Campus, Roosevelt Drive, Oxford OX3 7FZ, email: [daniel.ebner@ndm.ox.ac.uk](mailto:daniel.ebner@ndm.ox.ac.uk), phone: +44 1865 612923, ORCID 0000-0002-6495-7026

* Correspondence to [elena.di-daniel@astx.com](mailto:elena.di-daniel@astx.com) and daniel.ebner@ndm.ox.ac.uk


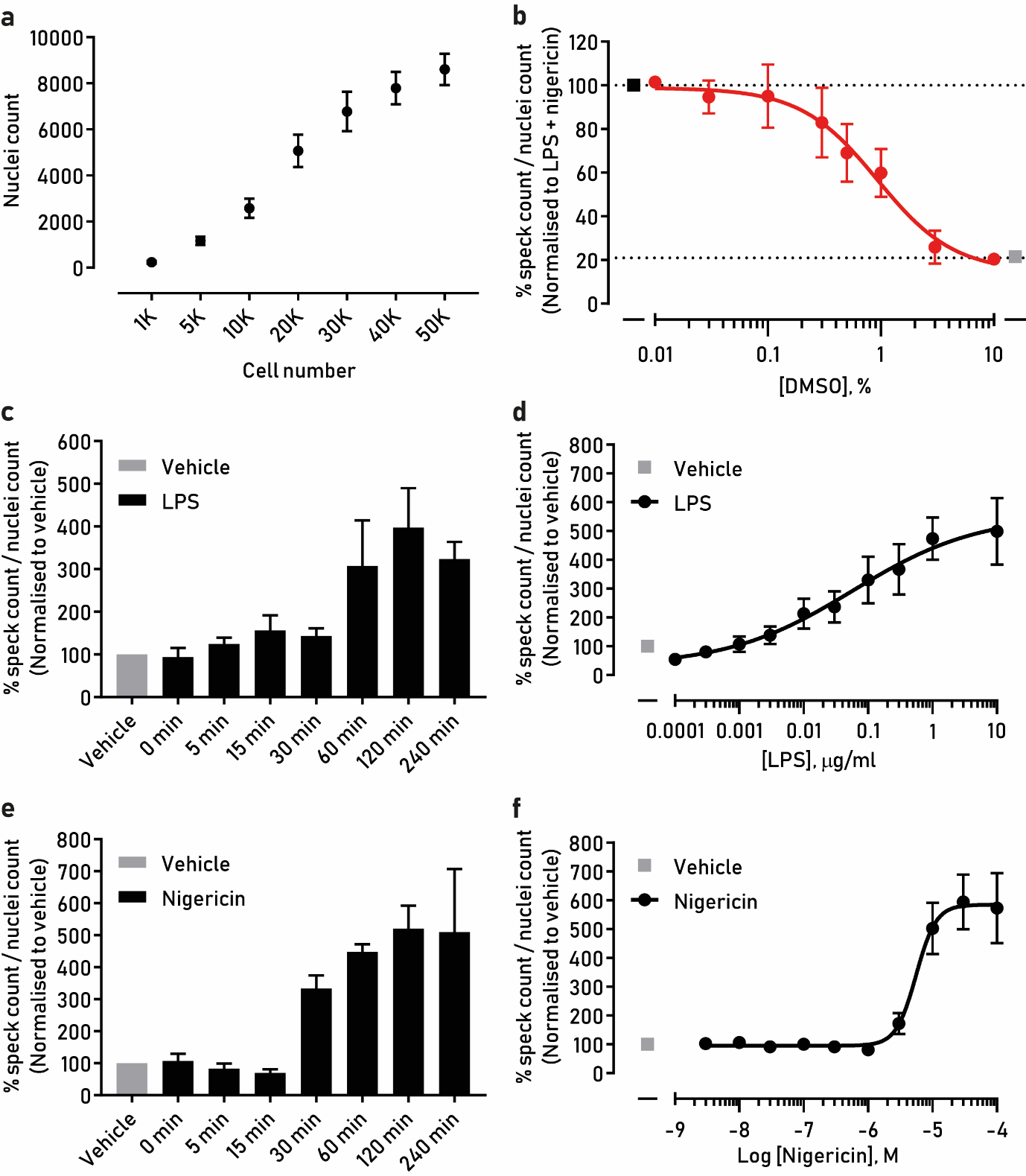


Figure S1 Speck assay optimization. (a) Different cell densities (1, 5, 10, 20, 30, 40 or 50,000 cells/well) of iBMDMs were seeded into 384-well plates, incubated with Hoechst (2 µg/ml) and imaged to identify the cell density resulting in a cell monolayer. 10,000 cell density was chosen and used in subsequent experiments. (b) DMSO assay tolerability was examined with indicated concentrations of DMSO incubated with LPS (1 µg/ml, 2 h) and subsequently stimulated with nigericin (10 µM, 2 h). Cells were fixed with PFA and specks counted. (c) iBMDM cells were incubated with LPS (1 µg/ml) for the indicated incubation periods and stimulated with nigericin (10 µM, 2 h) before speck counting. (d) iBMDM cells were incubated with LPS at indicated concentrations for 2 h followed by stimulation with nigericin (10 µM, 2 h), fixed with PFA and specks were counted. (e) iBMDM cells were stimulated with LPS (1 µg/ml for 2 h) followed by stimulation with nigericin (10 µM) for the indicated incubation periods, fixed with PFA and specks counted. (f) iBMDM cells were stimulated with LPS (1 µg/ml for 2 h) followed by nigericin at the indicated concentrations for 2 h, fixed with PFA and specks were counted. Data are presented as mean + SEM, n=3 independent experiments.


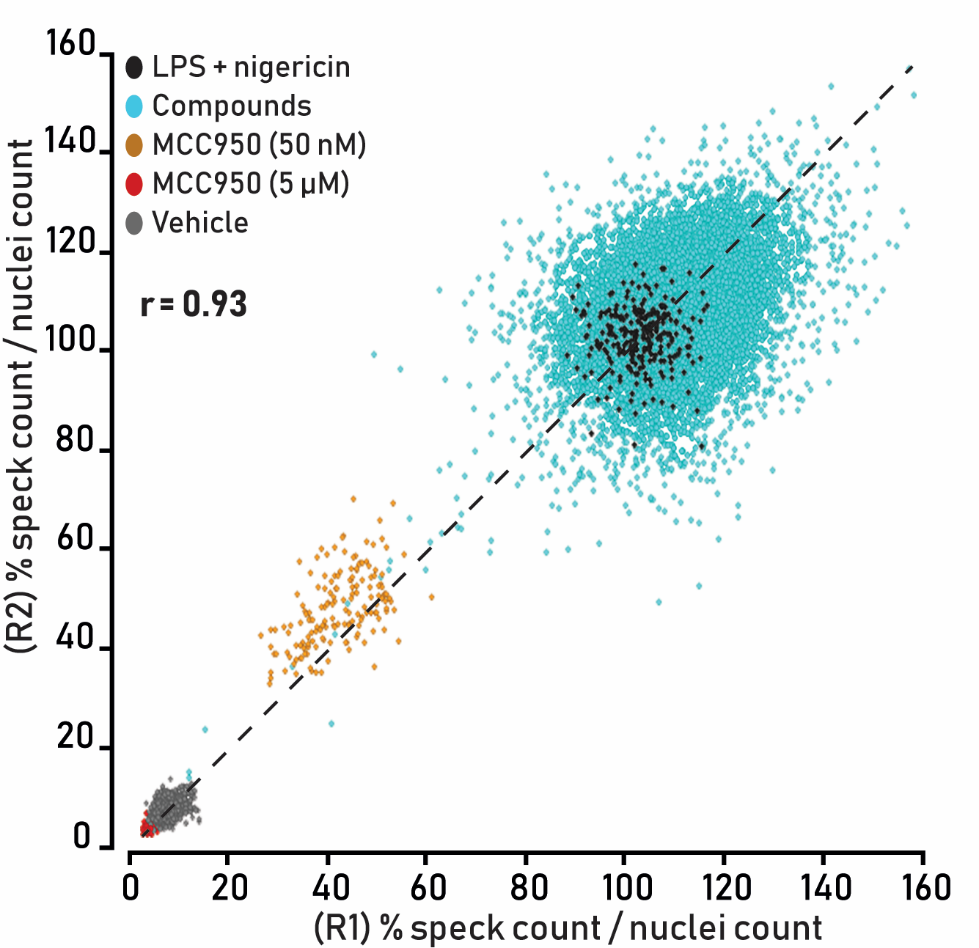


Figure S2 Replicate correlation data of first 10,000 compounds of the 81,000 compound library. 10,000 compounds were tested in two rounds (R1 and R2) and plotted R1 vs R2 showing an r value of 0.93. LPS + nigericin without compounds (black), LPS + nigericin in the presence of compounds (cyan), different concentrations of MCC950 at 50 nM (orange) and 5 µM (red) and vehicle (grey). Data presented are single points.


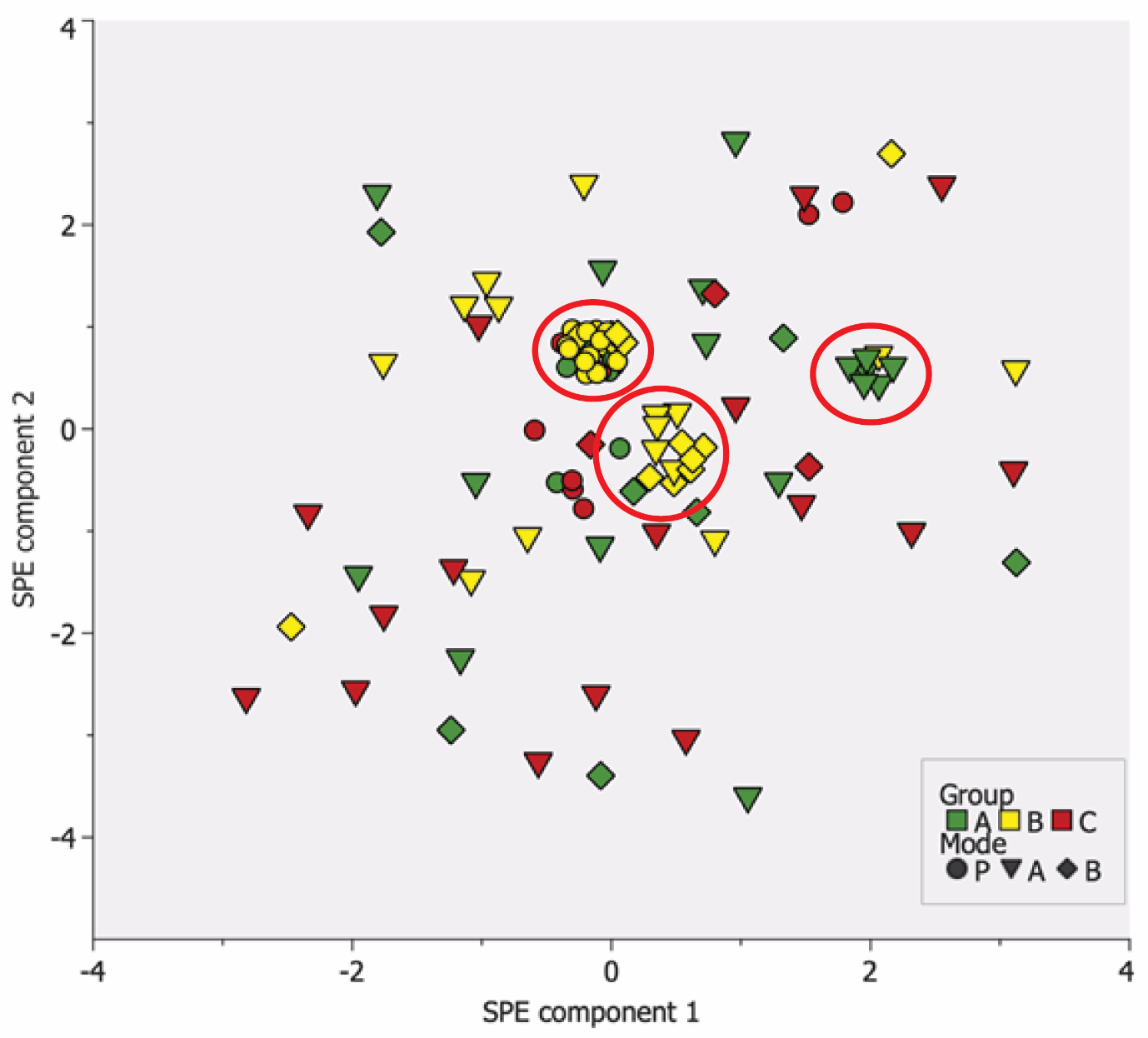


Figure S3 Compound hits profiled in the priming and activation protocols and compared for structural similarity. From amongst the hits, compounds were identified as strong hits with complete speck inhibition and with a Hill slope of 1.0 (group A), moderate hits with complete speck inhibition and with a non-standard Hill Slope (group B) and weak hits with a partial curve of at least 30% speck inhibition (group C). The hits are also annotated with different shapes depending on their effect on priming (circle), activation (inverted triangle) or indistinguishable (diamond). The axes are unitless components generated from a clustering of the compound chemical structures using fingerprint similarities and the stochastic proximity embedding (SPE) approach^42^. Three clusters are highlighted within the red circles.


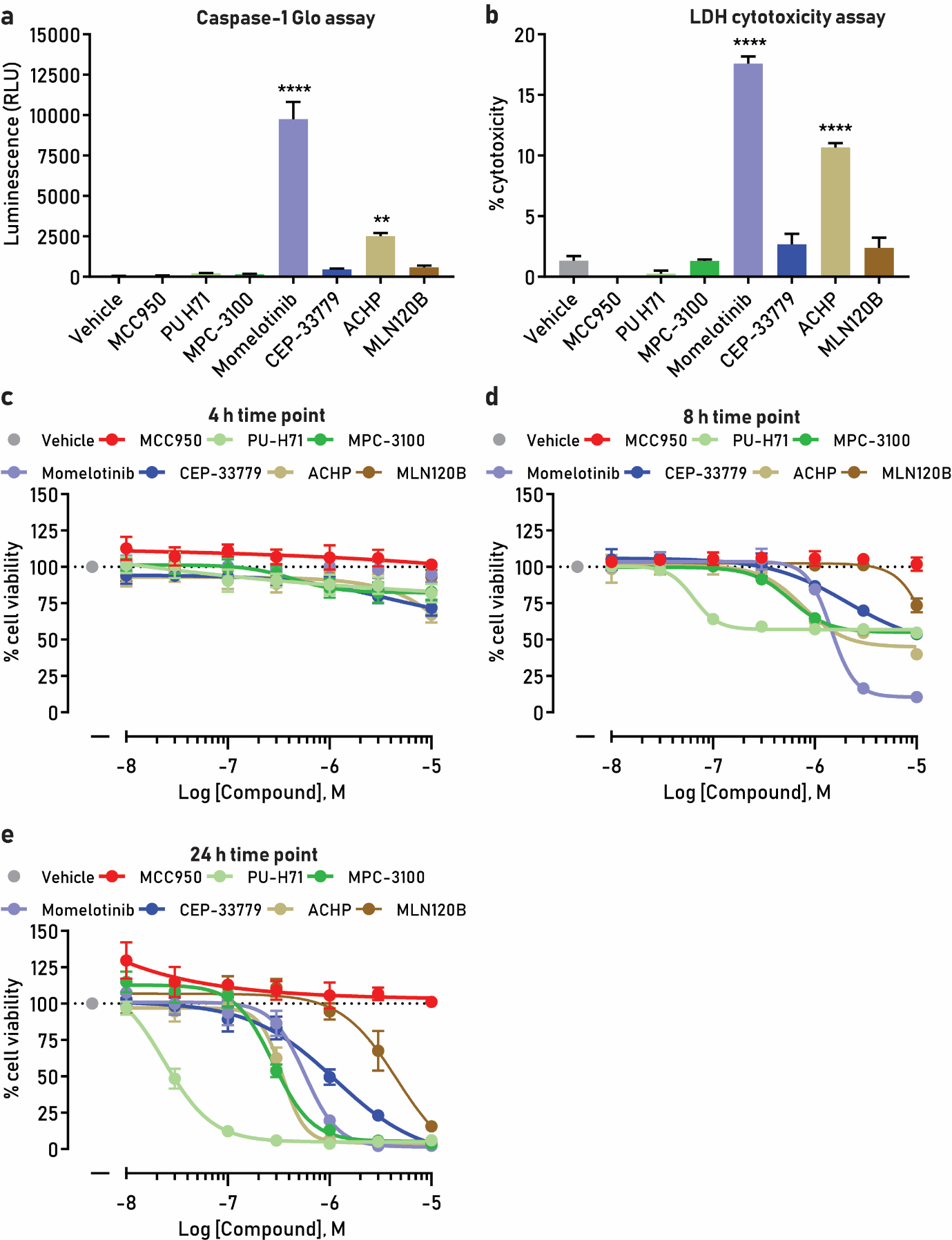


Figure S4 Cell viability of MCC950, HSP90, JAK and IKK-β inhibitors. Indicated compounds (10 µM) were incubated with iBMDM cells without LPS or nigericin. Cell supernatants were examined in (a) Caspase-1 Glo assay (4 h stimulation), (b) LDH assay (4 h stimulation), (c, d, e) CCK-8 assay (4 h, 8 h, 24 h stimulation, respectively). Mean + SEM, n=3 for all assays.

Figure 1D – IL-1β (p30 and p10)


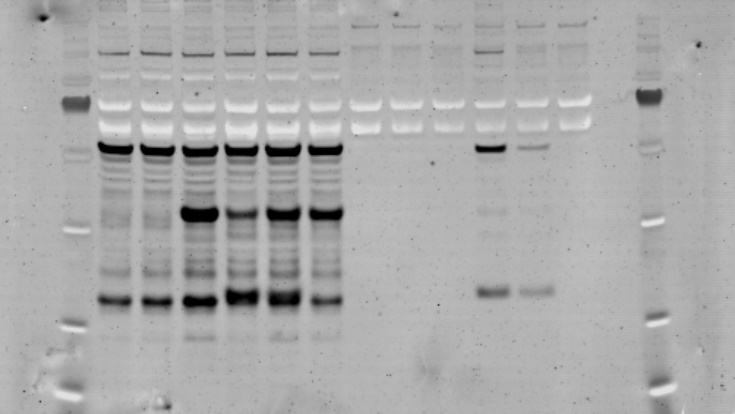


Figure 1D – IL-1β controls (β-Actin)


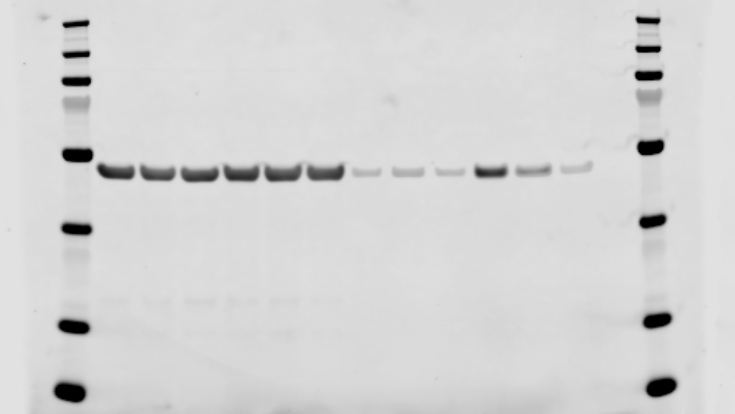


Figure 1D – caspase-1 (p45 and p20)


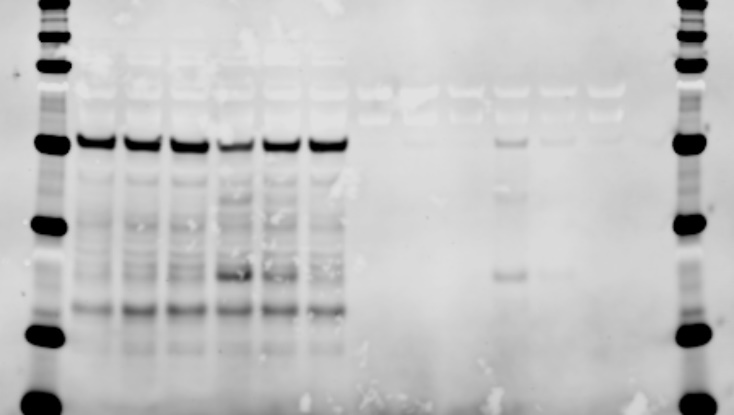


Figure 1D – caspase-1 controls (β-Actin)


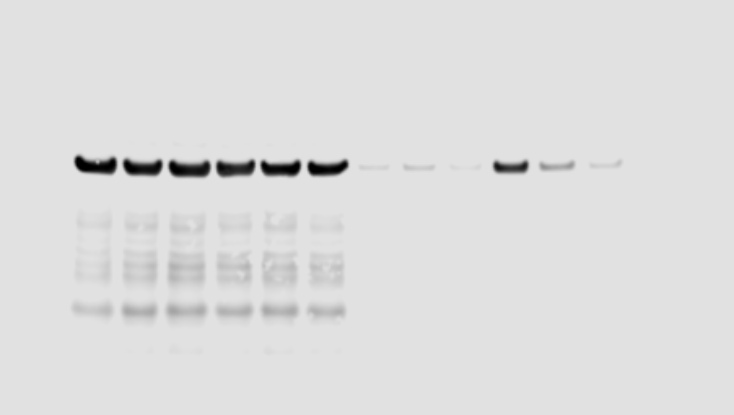


Figure S5 Full Western blots of caspase-1 and IL-1β. Cropped parts of bands of interest are shown in Figure 1D.

Figure 5A – Pro-IL-1β (p30)


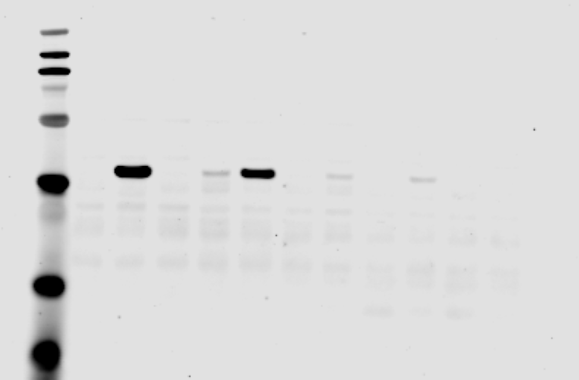


Figure 5A – IL-1β (p17)


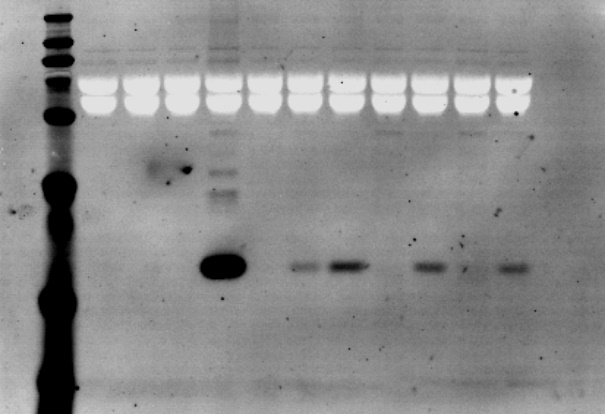


Figure 5A – IL-1β controls (β-Actin)


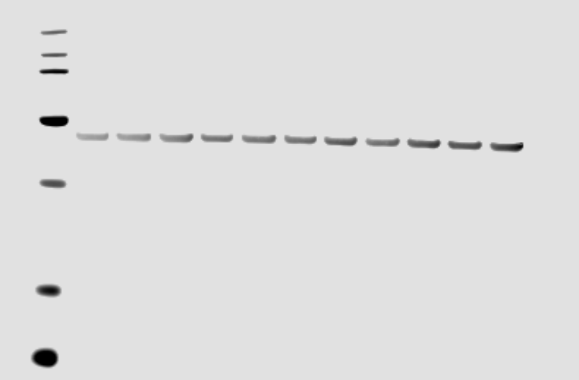


Figure 5A – caspase-1 (p45)


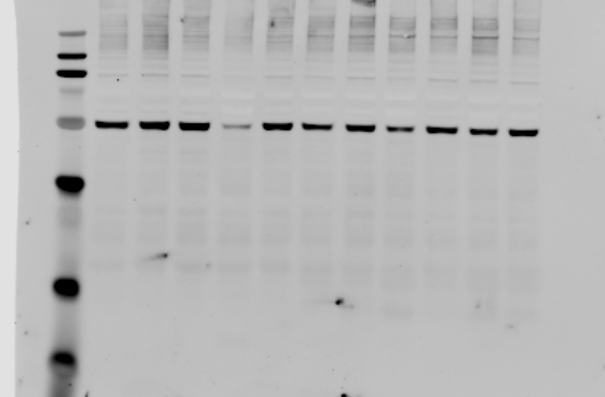


Figure 5A – caspase-1 (p20)


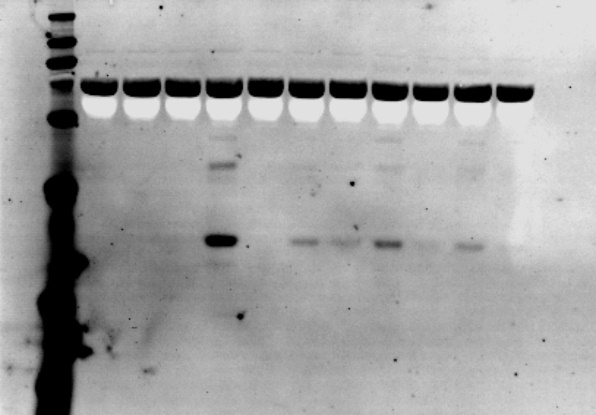


Figure 5A – caspase-1 controls (β-Actin)


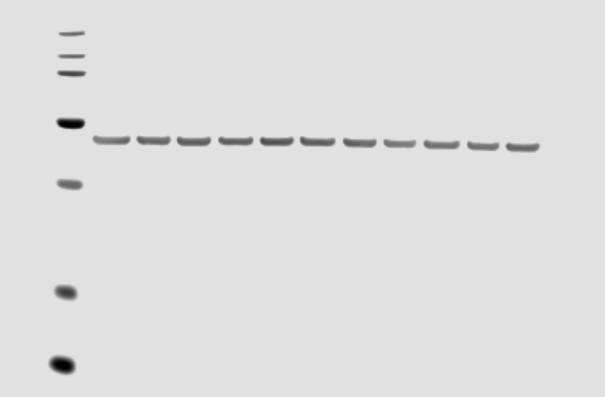


Figure S6 Full Western blots of caspase-1 and IL-1β. Cropped parts of bands of interest are shown in Figure 5A.

| **Plate Uniformity Assessment Validation Checklist** | **Meets criterion?** |
| --- | --- |
| **Intra-plate tests** | |
| Check for drift and edge effects in all plates (manually) | Yes |
| All LO signal CV < 20 % | Yes |
| All MID signal (unnormalized) CVs < 20 % | Yes |
| All normalized MID signal (Mid %) SD's < 20 | Yes |
| All HI SD's < Min (HI SD, MID SD) | No |
| All SW's >2 | Yes |
| All Z Factors > 0.4 (and < 1; must pass one of 6 or 7) | Yes |
| **Inter-plate tests** | |
| All within-day fold shifts < 2 | Yes |
| All average (between-) day fold shifts < 2 | Yes |
| **Ratio EC_50_/IC_50_/Ki** | |
| Ratio EC_50_/IC_50_/Ki within days (larger over smaller) | Yes |
| Ratio EC_50_/IC_50_/Ki between days (larger over smaller) | Yes |

**Table S1 Plate uniformity assessment validation checklist**. The table shows the validation checklist of the plate uniformity assessment data in which robustness of the assay was assessed using the NIH protocol of 3 plates over days^24^. The assay passed the criteria set within the template of the protocol. LO = low assay signal (no specks), MID = mid-point of assay signal and HI = high assay signal (max specks).

|  | Edge wells data not included | | | Edge wells data included | | | Whole Plate Metrics | | | |
| --- | --- | --- | --- | --- | --- | --- | --- | --- | --- | --- |
| Statistical Parameters | Mean | SD | CV | Mean | SD | CV | Z’ factor | Signal Window | Max/Min Ratio | Spec (%) |
| Day 1 plate 1 | - | - | - | - | - | - | 1 | 169 | 8.7 | 88.5 |
| Max | 50.1 | 0.3 | 0.5 | 49.2 | 0.3 | 0.5 | - | - | - | - |
| Mid | 37.7 | 2.9 | 7.6 | 37 | 3.3 | 9 | - | - | - | - |
| Min | 6.3 | 0.3 | 4 | 5.7 | 0.3 | 4 | - | - | - | - |
| Day 1 plate 2 | - | - | - | - | - | - | 0.8 | 14.4 | 32.4 | 96.9 |
| Max | 49.6 | 2 | 4.2 | 49.1 | 2.7 | 5.5 | - | - | - | - |
| Mid | 38 | 2.1 | 5.6 | 36.7 | 3.4 | 9.3 | - | - | - | - |
| Min | 1.5 | 0.3 | 16.3 | 1.5 | 0.3 | 16.4 | - | - | - | - |
| Day 1 plate 3 | - | - | - | - | - | - | 0.8 | 14.2 | 33.7 | 97 |
| Max | 48.7 | 2.5 | 5.1 | 48.2 | 2.7 | 5.5 | - | - | - | - |
| Mid | 35.2 | 2.7 | 7.6 | 34.4 | 3 | 8.9 | - | - | - | - |
| Min | 1.4 | 0.2 | 15 | 1.4 | 0.3 | 19 | - | - | - | - |
| Day 2 Plate 1 | - | - | - | - | - | - | 0.8 | 12.6 | 26.7 | 96.2 |
| Max | 46.5 | 2.5 | 5.4 | 45.9 | 2.8 | 6 | - | - | - | - |
| Mid | 33.3 | 2 | 6.2 | 32.8 | 2.7 | 8 | - | - | - | - |
| Min | 1.7 | 0.3 | 16.6 | 1.7 | 0.4 | 22 | - | - | - | - |
| Day 2 Plate 2 | - | - | - | - | - | - | 0.8 | 18.8 | 22.6 | 95.6 |
| Max | 45.7 | 1.6 | 3.5 | 45.3 | 2 | 4.3 | - | - | - | - |
| Mid | 33.5 | 1.8 | 5.4 | 32.4 | 2.7 | 8.4 | - | - | - | - |
| Min | 2 | 0.3 | 13.9 | 2 | 0.3 | 14.9 | - | - | - | - |
| Day 2 Plate 3 | - | - | - | - | - | - | 0.7 | 8.9 | 19.9 | 95 |
| Max | 43 | 3 | 6.9 | 42.7 | 3.3 | 7.6 | - | - | - | - |
| Mid | 29.9 | 3.4 | 11.4 | 30.3 | 4.1 | 13.6 | - | - | - | - |
| Min | 2.1 | 0.3 | 16.5 | 2.1 | 0.6 | 26.8 | - | - | - | - |

**Table S2 Plate uniformity assessment metrics**. The table shows the metric data from the plate uniformity assessment experiment in which robustness of the assay was assessed using the NIH protocol of 3 plates over 2 days^24^. Data shows the comparison of the mean, standard deviation and CV values of the six plates not including and including edge wells of the plate and the overall Z’ factor, signal window, max/min ratio and spec (%) of the whole plate.
